# Microstructure visualization of conventional outflow pathway and finite element modeling analysis of trabecular meshwork

**DOI:** 10.1186/s12938-016-0254-2

**Published:** 2016-12-28

**Authors:** Jing Zhang, Lin Ren, Xi Mei, Qiang Xu, Wei Zheng, Zhicheng Liu

**Affiliations:** 1 School of Biomedical Engineering, Capital Medical University, Beijing, 100069 China; 2 Beijing Key Laboratory of Fundamental Research on Biomechanics in Clinical Application, Capital Medical University, Beijing, 100069 China; 3 Shenzhen Key Lab for Molecular Imaging, Research Lab for Biomedical Optics and Molecular Imaging, Shenzhen Institutes of Advanced Technology, Chinese Academy of Sciences, Shenzhen, 518055 China

**Keywords:** Conventional outflow pathway, Trans-scleral imaging method, 3D reconstruction, Porosity, Finite element modeling

## Abstract

**Background:**

The intraocular pressure (IOP) is maintained through a dynamic equilibrium between the production and drainage of aqueous humor. Elevation of intraocular pressure is mainly caused by the blocking of aqueous humor outflow pathway. Therefore, it is particularly important to study the structure of drainage pathway and the effect of ocular hypertension at the process of aqueous humor outflow.

**Methods:**

Conventional drainage pathway of aqueous humor, including trabecular meshwork (TM), Schlemm’s canal (SC) and aqueous vein, were imaged by using trans-scleral imaging method with lateral resolution of 2 μm. For quantitative assessment, the morphological parameters of the TM were measured with different IOP levels via a combination of measurements and simulations.

**Results:**

Images of the TM and the adjacent tissues were obtained. The porosity of TM with normal intraocular pressure varies from 0.63 to 0.74 as the depth increases, while in high IOP it is changed from 0.44 to 0.59. The diameter of aqueous vein varies from 32 to 43 μm, and is smaller than that of SC, which varies from 48 to 64.67 μm.

**Conclusions:**

Our research provides a non-contact method to visualize the microstructure of tissue for clinical examination associated with the blocking of the outflow pathway of aqueous humor in humans. The three-dimensional (3D) microstructures of limbus and the results of finite element modeling analysis of the TM model will serve for the future evaluation of new glaucoma surgical techniques.

## Background

Glaucoma, which can result in the damage of optic nerve and defect of visual field, is thought to be the highest irreversible blinding eye disease in the world [1, 2]. Elevation of intraocular pressure (IOP) mainly caused by the blocking of aqueous outflow system is a high risk factor for glaucoma [3]. Aqueous humor leaves the eye mainly from conventional outflow pathway which contains the trabecular meshwork (TM), Schlemm’s canal (SC) and collector channels (CC) before finally draining into aqueous vein. The aqueous humor flow through the TM is driven by a pressure gradient across the tissue. Thus, the pressure difference between inside (intraocular pressure, IOP) and outside (episcleral venous pressure, EVP) provides the force to make aqueous humor flow out of the TM [4]. During primary open angle glaucoma, the greatest resistance to aqueous humor flow is provided by the morphological changes in TM [5–7]. To clarify the production of outflow resistance, it is necessary to obtain the microstructure of drainage pathway.

Consequently, there is a high demand for an effective method to reveal the detailed structure of trabecular meshwork and adjacent tissues. There are several techniques used to inspect ocular tissues. As one of the frequently-used clinical diagnostic equipment, optical coherence tomography (OCT) has a resolution of 15–25 μm [8], while the light scattering properties of sclera causes imaging blur. Another common technique is ultrasound biomicroscopy (UBM) [9]. The limitations of these two technologies lead to the low resolution of image. It remains unknown about three-dimensional (3D) microstructure of aqueous humor outflow pathway in physiological state. Besides that, because of the very tiny size of tissues in the aqueous outflow system, the detailed 3D information of trabecular meshwork tissues is difficult to be detected by them. Two-photon microscopy (2PM) techniques, including two-photon autofluorescence (2PAF) and second harmonic generation (SHG), which have been widely regarded as a useful tool for 3D imaging, have advantages on depth of field and lateral resolution [10–12]. Meanwhile its high penetration rate can be used for deep tissue imaging with long wavelength excitation. And the long wavelengths of the infrared lasers used in 2PM have considerably less chance to thermally damage [13]. Zoumi et al. showed that 2PM can be used to excite endogenous biological chromophores such as elastin/collagen and melanin [14]. TM with component of collagen fibers and elastin could be imaged by 2PM, which is an effective method for imaging of the TM in its native state and can obviate histology distortion by the cause of fixation and histological processing. As TM locates in deep tissue, 2PM is an available imaging method to obtain image in situ.

In this paper, images of TM and its surrounding tissues were obtained simultaneously. Using these images to measure the size of SC and porosity of TM were particularly close to morphology. Comparing to the structure of 3D reconstruction using histology section, using optical tomography images to reconstruct the TM region of intact eye can get a structure which is based on physiological state. Microstructure under high IOP may be considered in clinical diagnosis with our 3D reconstruction results. 3D structures of TM in different intraocular pressure were reconstructed respectively, which can be used to get the deformation field of the TM after finite element modeling analysis. Moreover, analysis and comparison are made between the results of the morphologic changes of TM from the experiment and numerical simulation. The findings in this report would make sense for a better understanding and wider applications of diagnosis and therapy of glaucoma.

## Methods

### Rat eye tissue

Six SD rats with no ocular phenotype were provided by the animal department (Capital medical University, Beijing, China, IACUC: AEEI-2013-x-123). The weights of the rats were between 300 and 350 g. After euthanasia, we divided them into two groups. Group A (n = 3): eyes were enucleated and held in phosphate-buffered saline (PBS; 8 g/L sodium chloride, 0.2 g/L potassium phosphate monobasic, 2.16 g/L sodium phosphate dibasic heptahydrate, pH 7.4) for intact eye imaging. Group B (n = 3): eyes were enucleated and preserved in 4% paraformaldehyde for 12 h at 4 °C for fixation, and then washed in PBS. After that, anterior segments consisting of cornea, anterior sclera, and trabecular meshwork with iris were prepared by cutting along the equator of the globe and placed in a culture dish with the TM side up for broken eye imaging.

### Two-photon microscopy imaging

**Fig. 1:**
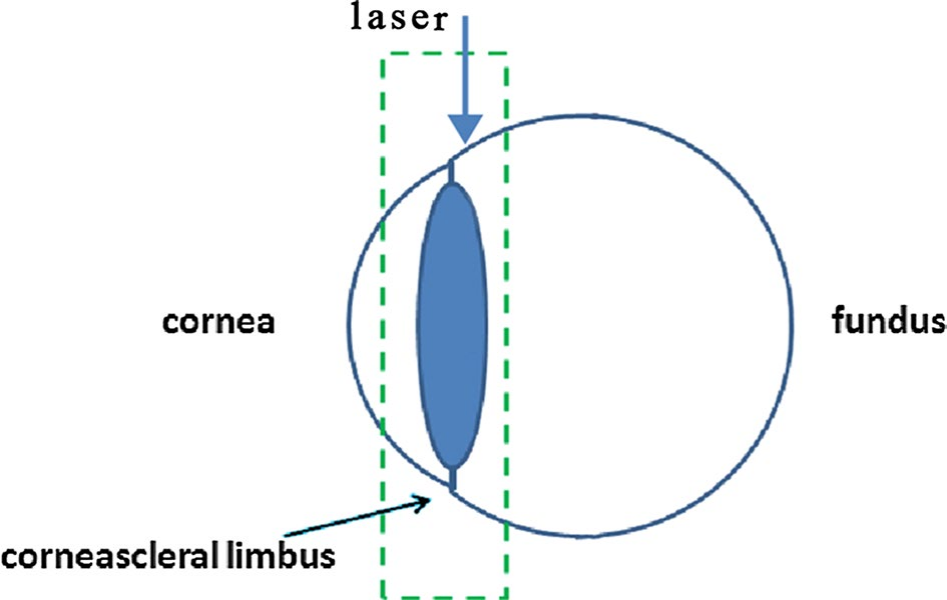
The laser exposed surface of eye from limbus vertically, sequential images with step length of 5 μm were then collected through the trans-scleral imaging. *Green dotted box* corresponds to the imaging area

The two-photon microscopy system set up by Shenzhen Institutes of Advanced Technology [15], Chinese Academy of Sciences was equipped with a tunable mode-locked Ti: Sapphire laser (Chameleon Ultra and Chameleon Vision Laser; Coherent Inc., Santa Clara, CA) operating at 800 nm center wavelength, with 140 fs pulses at 80 MHz repetition rate. The excitation source (Ti: Sapphire laser) was focused on the tissue samples by a XLUMPLFLN 20X/1.00 NA objective with a 2 mm working distance (Olympus Inc.). Firstly the emitted signal passed through a FF01-680/SP-25 filter (semrock Inc) to remove residual excitation laser light, then went through ET402/15× filter for SHG signal detection. The signal was detected by PMT (H742-50, Hamamatsu Photonics Co.).The Labview is the software which collects and processes the image stacks and controls all devices. Figure 1 schematically shows the image region. The image size is 512 × 512 pixels, and the resolution is 2 μm.

### Image analysis

**Fig. 2:**
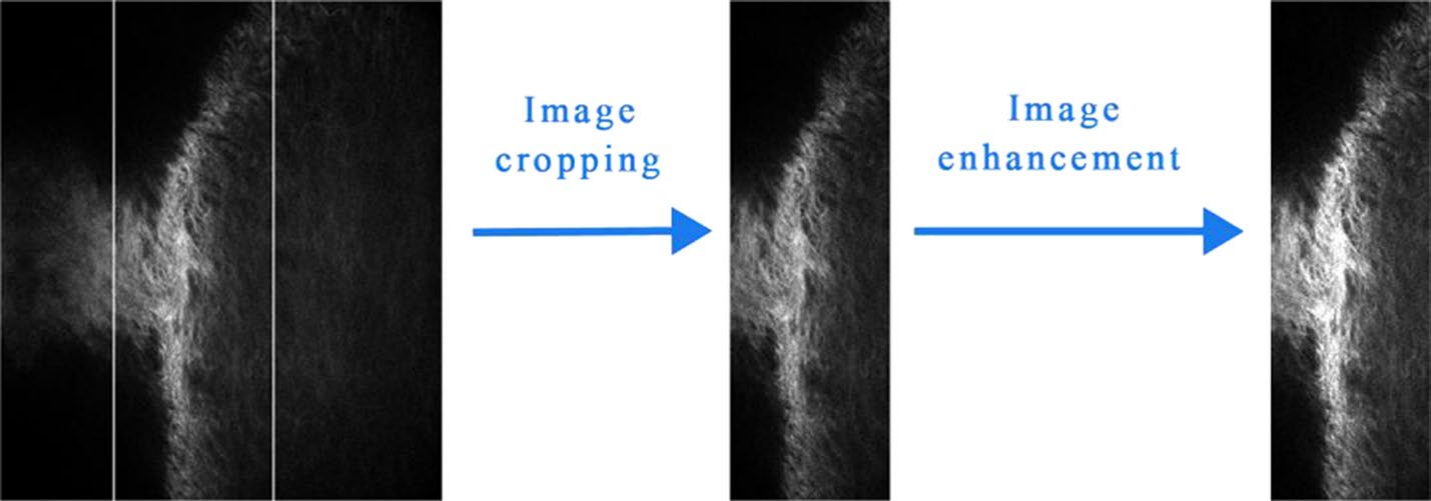
Program flow chart of image process before porosity calculation. The *left region* of image is cornea and the *right region* is sclera

Figure 2 shows the image process. We firstly cropped the limbus from the image to obtain valid image, then enhanced the image and segmentation to acquire porosity. For calculation of porosity, we get segmentation threshold from gray histogram and calculated the ratio of pores’ area in valid image. The sizes of SC in the original images of intact rat eyes were measured with different depths. For each eye, we measured the size of the tissue for five times at different depths, then averaged the data to get the mean size of SC.

### Modeling

The entire stack of images from the 2PM was used to reconstruct the TM structures. The format of the images of TM is transformed into software MIMICS10.0 ((Materialise Inc., Leuven, Belgium). In this study, the images are processed using the tools of threshold segmentation, region glowing and edit. Then, the three remodeling model of TM region can be obtained.

### Boundary condition

The TM and sclera materials were assumed to be linearly elastic and isotropic. The simulated pressures were set to the pressure drop between IOP and EVP in the rat eye. The material properties of TM model were a Poisson ratio of 0.48 and Young’s modulus of 162 Pa [16]. The simulated pressures were set from 10 to 160 Pa with an interval of 10 Pa. The direction of pressure is perpendicular to the plane in parallel to the inner wall of SC. Pressure was applied on the TM and the sclera edge was set as the constraint for the analysis of pressure drop across the TM tissue.

## Results

### Trabecular meshwork imaging and porosity

**Fig. 3:**
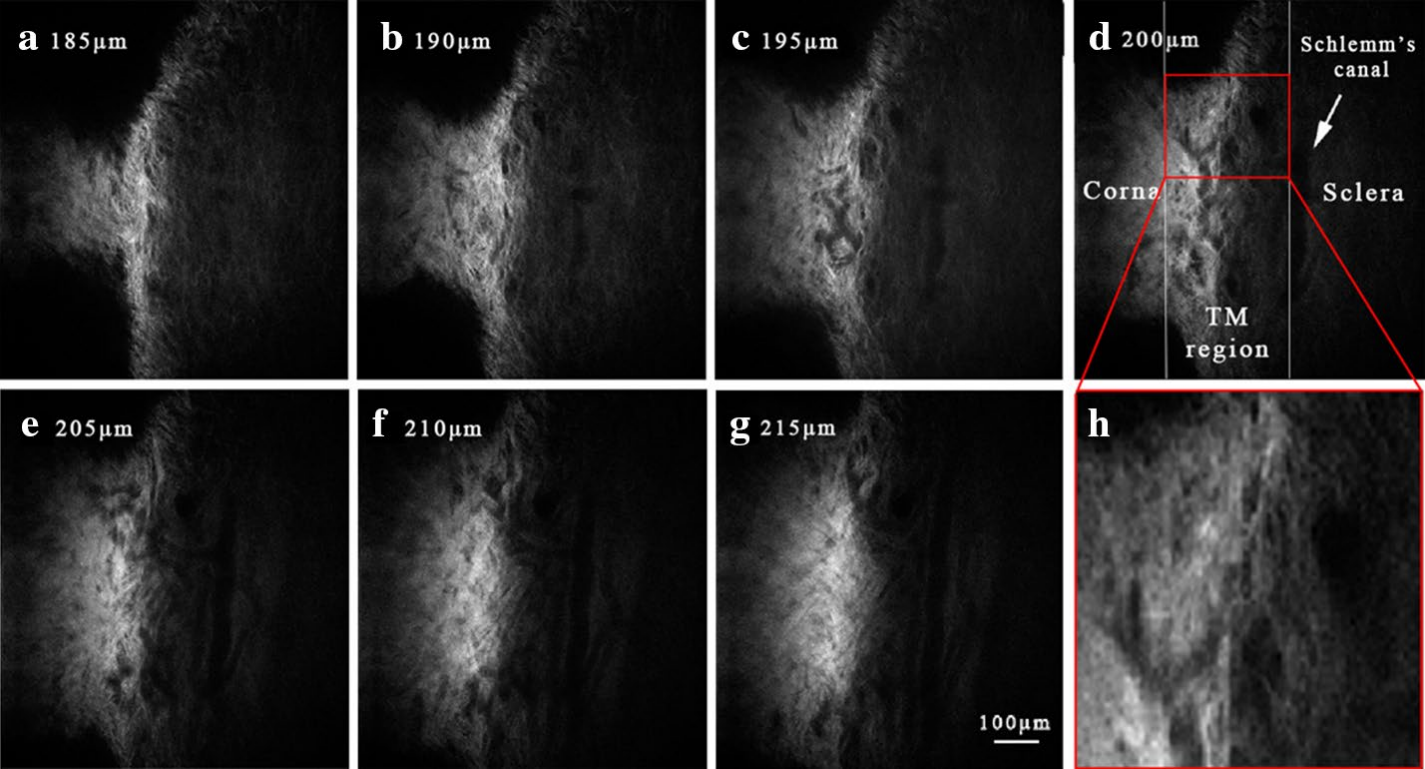
**a**–**g** SHG images of the TM region in an intact rat eye at different depths (from 185 µm to 215 µm with an interval of 5µm). As surface of eye was set to be 0 μm, **a** is the image closest to the surface and **g** is the image close to the innermost regions of TM. **h** is the image of amplified the *red box* in **d** with four times. *Scale bar* 100 μm

Figure 3a–g illustrated SHG images at different depths within the TM region from 185 µm beneath the superficial surface of eye to 215 µm with an interval of 5µm. According to the location of imaging, the left region is cornea and the right region is sclera. From these images, we can see that areas lacking signals are corresponding to the fluid-filled gaps, and the gaps seem to be heterogeneous with the depth increases. After amplified red box region in Fig. 3d for four times and shown in Fig. 3h, several small pores were found between fiber tissues, and this region was consistent with the anatomical position of trabecular meshwork.

**Fig. 4:**
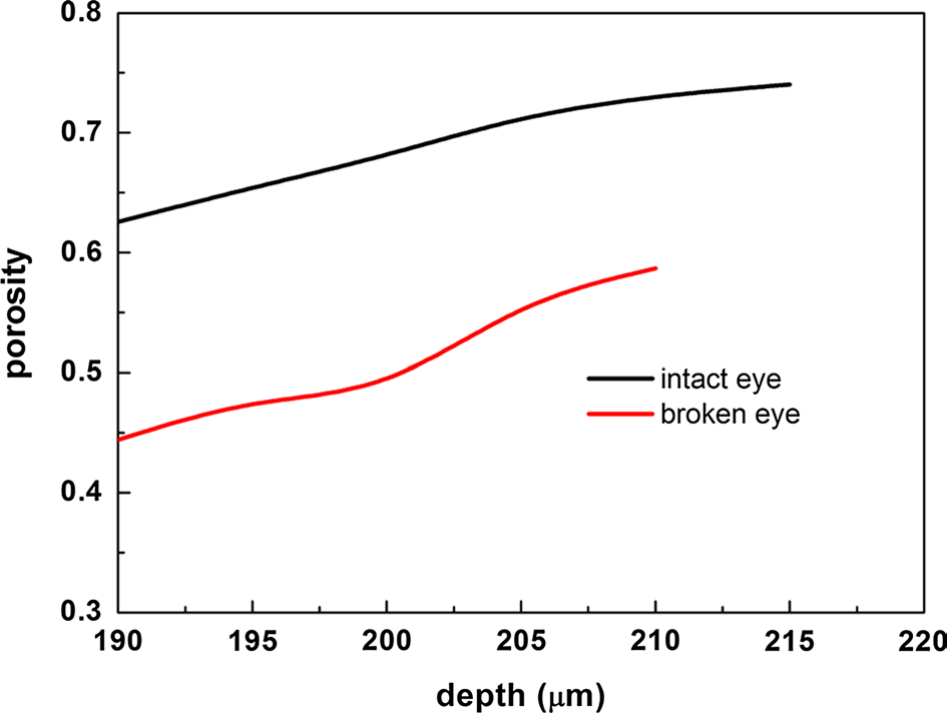
Porosity in different depths of TM region in intact rat eye (*black solid line*) and broken rat eye (*red solid line*)

The graph of relation between porosity and the depth of TM in intact rat eye and broken rat eye are shown in Fig. 4. As the 2PM imaging depth increased, porosity of intact eye became larger and varied from 0.63 to 0.74. It can be easily found that the porosity of TM in broken eye was smaller than that in intact eye. It is suggested that the TM tissue collapsed and fused into surrounding tissues as a result of the maintenance of discharged IOP in the broken rat.

### Schlemm’s canal imaging

**Fig. 5:**
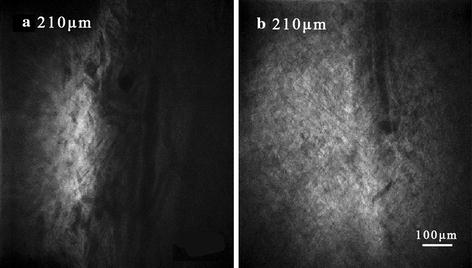
Images of SC from the same depth in intact eye (**a**) and broken eye (**b**). *Scale bar* 100 μm

Some researchers located SC within postmortem primary tissue by identifying a broad circumferential autofluorescence signal void in serial optical sectioning just beyond the final cellular layer of the TM [17]. As we can see from Fig. 5a, a canal without 2PAF appears below the sclera. The depth of the images was measured from 190 to 215μm. Roughly speaking, the average size of the canal varies from 40 to 64 μm. According to the anatomical position and size of the channel-like zone, the canal was supposed to be the oval SC.

**Fig. 6:**
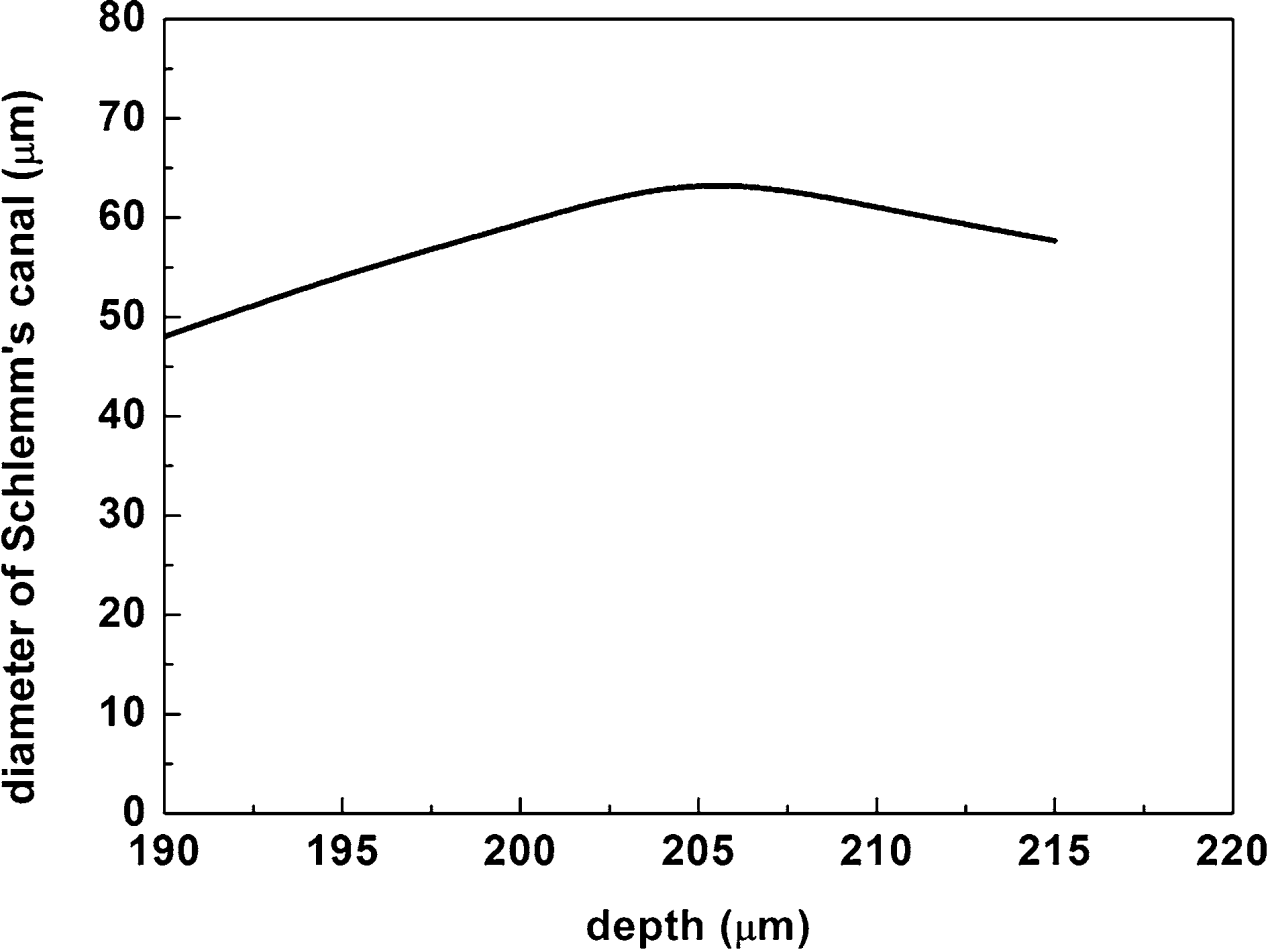
The graph of relation between the average diameter of Schlemm’s canal and the depth of tissue in intact rat eye

Schlemm’s canal is approximate oval shape around the limbus and the diameter of SC varies with tissue depths. We measured the mean size of SC in different depths of tissue (see Fig. 6). The result shows that the diameter of the SC varies from 48 to 64.67 μm as the imaging depth of the 2PM system increases. And by contrast with the data measured in other depths, there is a maximal value of SC with a value of 64.67 μm at the depth of 207 μm .

## 3D reconstruction of TM and Schlemm’s canal

**Fig. 7:**
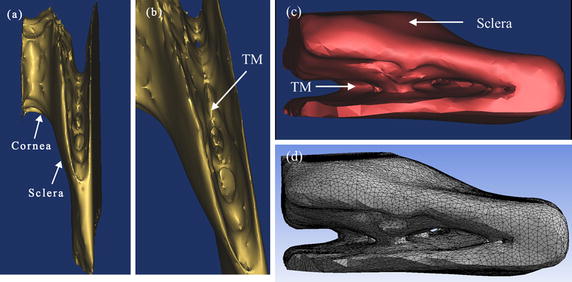
**a** 3D limbus model of broken rat eye. **b** Partial enlargement drawing of **a**. **c** 3D remodeling model of the TM in intact rat eye. **d** The TM model after the meshing

We also performed 3D reconstructions of optical section images to analyze the structure of limbus. 3D models of the TM region were reconstructed at normal and high IOP respectively, which are showed in Fig. 7. What can be seen from Fig. 7a is that the outside surface of sclera and cornea appears smooth. In the region of TM, just below the limbus, as shown in Fig 7 b, the TM also has relatively large pores which appear to be more functional for permitting aqueous humor flow fluently. There are some small pores fusing into the big pore. Structural feature of 3D TM model is consistent with TM configuration obtained by quick-freeze deep-etch electron microscopy [18].

### Morphological analysis of TM

Although analysis based on experiment has contributed to the understanding of the morphological structure of the TM, the detailed structural information of TM during the progression of intraocular pressure is not well-known. For the requirement of analysis, 3D finite element method (FEM) analysis on TM model was carried out using ANASYS 15. In the FEM analysis, if the TM model was combined with the sclera, TM biomechanical properties with more constraints could be acquired, resulting in a more accurate model. On this basis, 3D TM models included a 3D sclera model in the rat eye. The entity model of TM was processed by meshing to fit in FEM. As the final assembled model, shown in Fig. 7c, is the 3D remodeling model of TM. As can be seen in Fig. 7d, is the TM model of intact eye after the meshing, it was tested with the 193,566 elements and 575,076 nodes.

**Fig. 8:**
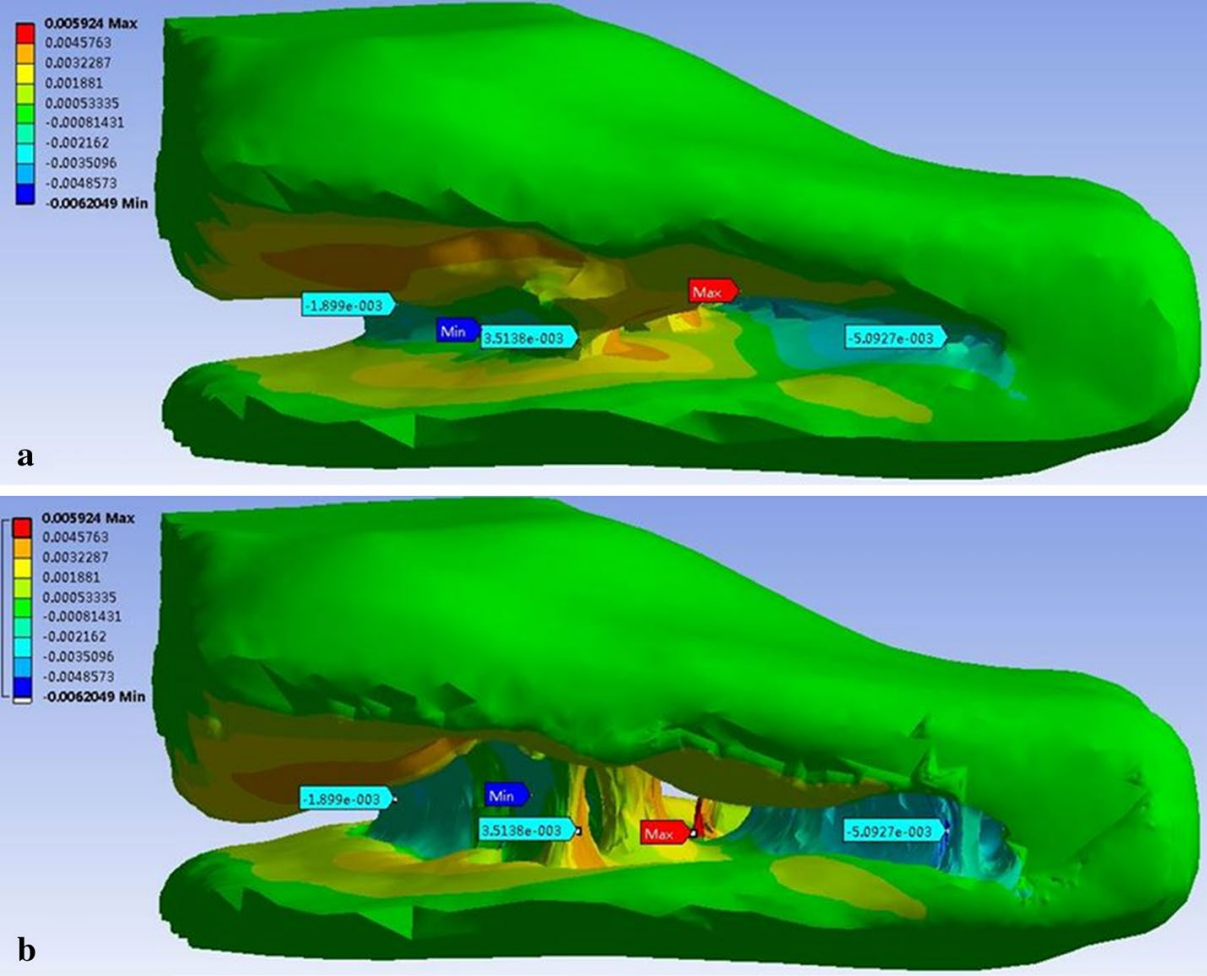
Distributions of the directional deformation of the TM model after the finite element modeling analysis with the simulated pressure in 60 Pa. **a** The directional deformation of the TM model. **b** The real deformation of the TM model

**Fig. 9:**
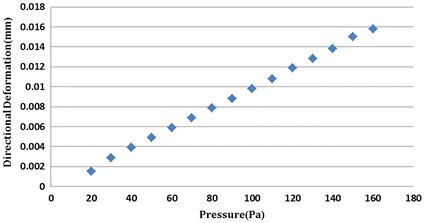
The values of the biggest directional deformation of TM model at different simulated pressures

From Fig. 8, the morphological changes of TM model and the large deformation points can be seen (red mark). The biggest area of deformation was located at a slender beam of TM. Meanwhile it is obviously accompanied with destruction on surface of trabecular beams. Also, the analysis results were compared with real changes of collagen fibers observed in the experiment. Figure 8a gives the details of deformation of TM with 60 Pa. All of these morphologic changes on internal area of TM are clearly seen from the Fig. 8b. In this way, the simulation results were in greater accordance with the real morphology of 3D model of the broken eye. As shown in Fig. 9, we obtained the directional deformation of TM in different pressures. We found that the greater the pressures was set, the higher damaged into the TM tissue and surrounding tissues. On the basis of previous studies (Fig. 4), the experimental results also indicate that the TM tissue collapsed and fused into surrounding tissues as a result of released IOP.

### Aqueous vein imaging and 3D reconstruction

**Fig. 10:**
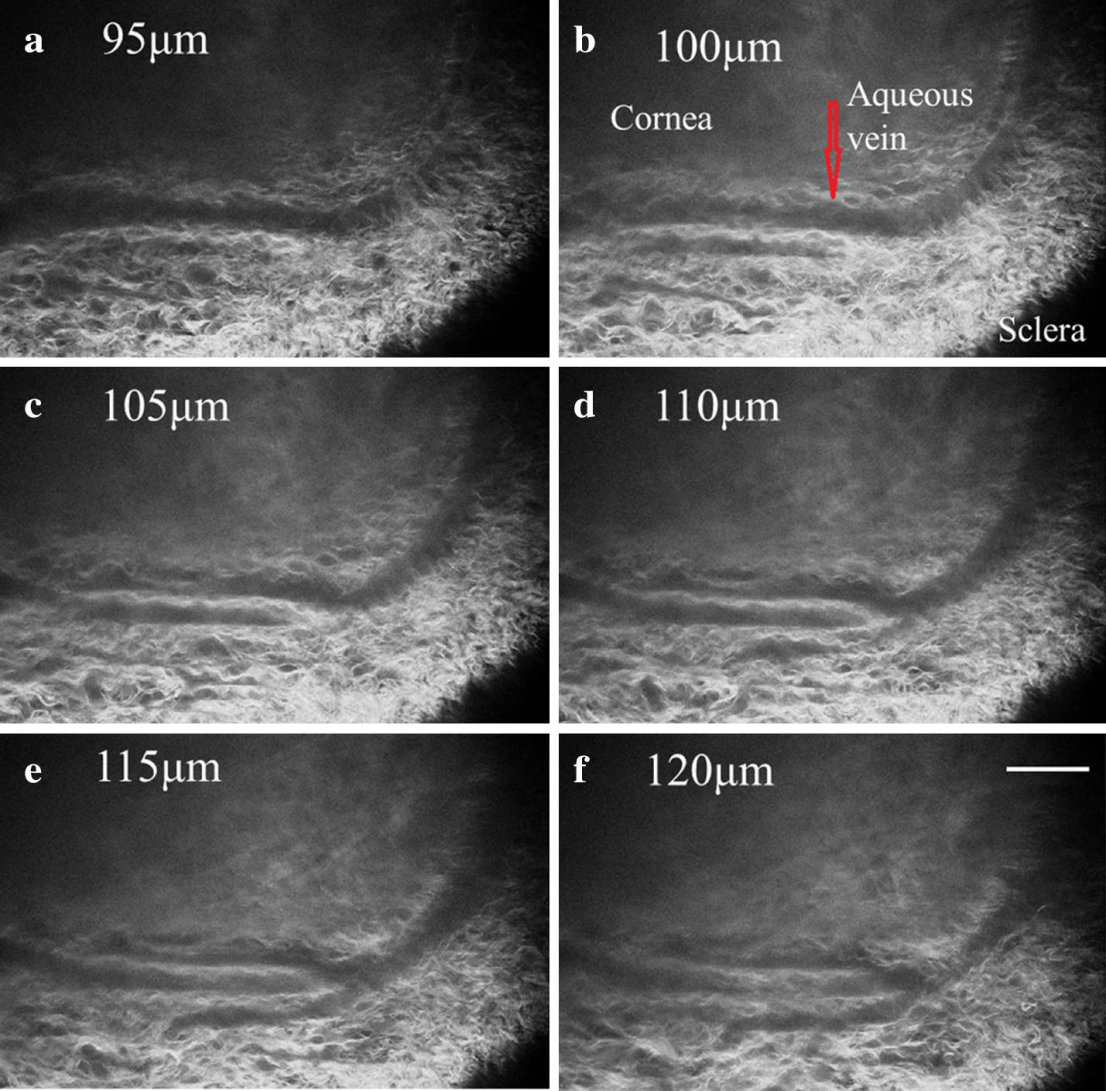
**a**–**f** SHG images of aqueous vein in intact eye at different depths (from 95 µm to 120 µm). As surface of eye was set to be 0 μm, **a** is closest to the surface and **f** is close to the innermost regions of TM. The imaging step is 5 μm. *Scale bar* 100 μm

To confirm the location of distal tissues in the trabecular drainage channels, we obtained SHG micrographs at different depths (1 μm apart) from the surface to the deep tissue. Figure 10 a–f shows the detailed structures of vessels and the peripheral tissues from 95 µm to 120 µm with an interval of 5 µm. The top and the bottom region of micrographs are cornea and sclera respectively. According to the anatomical position of branches, vessels in the micrographs are corresponding to the aqueous vein of which the diameter varies from 32 to 43 μm. Here, the size of aqueous vein is smaller than that of SC. Configuration of the aqueous vein appears to branch adjacent to the surface of eye.

**Fig. 11:**
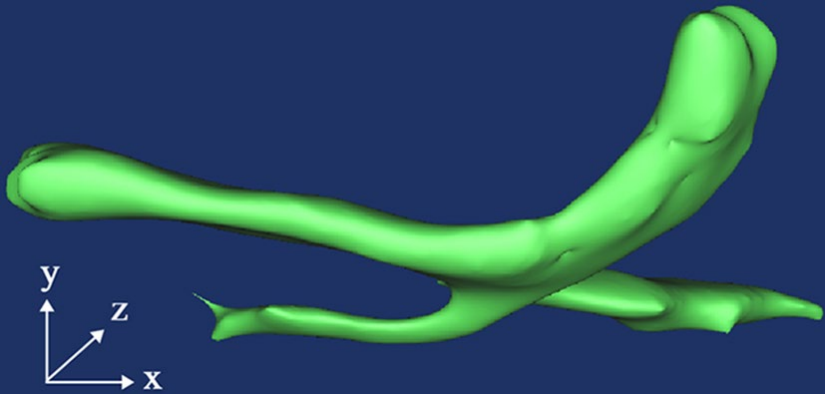
3D reconstruction of the aqueous veins in the intact rat eye

In order to get three-dimensional morphology of the aqueous vein, we reconstructed these serial images. Figure 11 displays the part of aqueous vein model. It is indicated that the aqueous veins widened to a flute shape at the point of anastomosis with the branch.

## Discussion

In our study, 2PM was used to image the trabecular drainage channels of intact enucleated rat eyes. Due to the intrinsic properties of drainage tissue, autofluorescence from collagen fibers can be used to image the microstructure of the pathway. Compared to external fluorescent label, autofluorescence can avoid tissue damage caused by fluorochrome. Meanwhile, 2PM is suited to tissue imaging under the sclera, and has been successfully used to image living skin at a depth of 350 μm by visualizing the 2PAF of the skin’s extracellular matrix and melanin [19]. Most importantly of all, we were able to successfully get the high resolution images under high scattering tissue of eye. Our imaging had a lateral resolution of 2 μm, and a depth resolution of 1 μm. It is found that the high resolution imaging can achieve resolution of tissue section, and can avoid tissue distortions caused by infusion of fixatives.

Aqueous humor drains into aqueous vein through TM region firstly. In this progress, TM and SC are both significant to fluid drainage. Obtaining the microstructures of these tissues is useful to investigate the production mechanism of outflow resistance in the whole draining tunnel. Imaging of TM in human cadaver eyes and obtaining the location of SC and TM in intact enucleated mouse eyes using 2PM has been reported respectively [20, 21].In this research work, we obtained the images of TM, the surrounding SC and aqueous veins totally. It also shows that rat and mouse eye tissue as a suitable animal model is useful for tussue imaging in the outflow pathway of aqueous humor.

Moreover, we calculated the porosity of TM in the intact eye and found that porosity became larger as depth increases. The porosity of TM in the broken eye was smaller compared to the intact eye. It can be seen that IOP plays an important role in the structure of TM. Force balance between IOP and episcleral venous pressure can maintain the physiological state of aqueous outflow pathway. Breaking the force balance can lead to the collapse of tissues in the trabecular drainage channels and then affect the IOP in return.

Some researches reconstructed limbus using histologic section with manual add CC to obtain the distribution of pressure and flow velocity in the model of eye and results offered were comparable to physiology references [22]. We also performed 3D reconstructions of serial images of limbus tissue to obtain the 3D structures of aqueous humor outflow pathway. From stereo image matching, we found that 3D TM model with some pores of various sizes was consistent with the form obtained from histologic sections. Which means that our 3D model based on ex vivo rat eye approaches to the physiology state, and can be used to simulate the physiological flow in TM region. Such observations of TM are interesting and important with pressure dependent changes in the trabecular meshwork.

There are several problems to be solved in the next step. Depth of penetration decreased image resolution due to scattering and tissue in homogeneities [23]. Previous research used gonioscopic lens to reduce highly scattering of tissue and obtain the structures of TM in the porcine eye [24]. The porcine eye has a series of CCs instead of a single large circumferential SC [25]. Compared with porcine eye, rat eye is smaller as well as the size of SC and aqueous veins [26, 27]. The resolution of image is the limitation of imaging quality in the drainage channels. Some researchers indicated that filtration patterns in human TM appeared segmental over both macro-scale and micro-scale dimensions [28]. To get a comprehensive description of aqueous humor outflow pathway, we will image the circumferential TM/SC/aqueous vein patterns instead of local regions. In order to provide sufficient evidence for diagnosis and therapy of glaucoma, accurately assessing structural changes of TM is very significant. To investigate the relation between the structural changes of limbus and the intraocular pressure, we performed 3D reconstructions of the TM region. In addition, static analysis of the deformation of a TM model was performed using the FEM. Moreover, the simulated results were combined with real changes obtained in our experiment. Early detection and clinical intervention in the prevention of glaucoma disease will greatly benefit from the images with high resolution and the results of deformation analysis. However, there are some limitations of the FEM analysis in this study. In the FEM analysis, if the TM model was combined with Schlemm’s canal and aqueous vein, TM biomechanical properties could be acquired, resulting in a more accurate model. To further modeling research, the model will be refined and the method will be improved, which may bring the hardness to interpret in terms of pathology. Potentially, it will become a valuable method for finding the resistance to drainage of aqueous humor and comprehensive assessment of treatment efficacy.

## Conclusion

Trans-scleral imaging method was used to obtain morphology of trabecular drainage channels, including TM/SC/aqueous vein, in the intact eye at different depths without staining and fixing. We also reconstructed the tissues of the drainage pathway to get 3D morphology and structure. Furthermore, we simulated and analyzed the structural changes of TM with different IOP levels by the FEM analysis. Combining the experiment results and simulations, our research provides a reliable method to visualize aqueous humor outflow pathway and a potential technique for clinical examination associated with the blocking of aqueous outflow pathway in humans.
